# A comprehensive perspective on the interaction between gut microbiota and COVID-19 vaccines

**DOI:** 10.1080/19490976.2023.2233146

**Published:** 2023-07-11

**Authors:** Ming Hong, Tin Lan, Qiuxia Li, Binfei Li, Yong Yuan, Feng Xu, Weijia Wang

**Affiliations:** a Institute of Advanced Diagnostic and Clinical Medicine, Zhongshan City People’s Hospital, Zhongshan, China; b Department of Anesthesiology, Zhongshan City People’s Hospital, Zhongshan, China; c Department of Cardiovascular Center, Zhongshan City People’s Hospital, Zhongshan, China; d Centre for Heart Lung Innovation, St Paul’s Hospital, University of British Columbia, Vancouver, BC, Canada

**Keywords:** COVID-19, vaccine efficacy, gut microbiota, immune responses, microbiota-targeted interventions

## Abstract

The efficacy of COVID-19 vaccines varies between individuals and populations, and the reasons for this are still not fully understood. Recent clinical studies and animal models have indicated that the gut microbiota may influence the immunogenicity of the vaccine and, thus, its effectiveness. This suggests that there is a bidirectional relationship between the gut microbiota and the COVID-19 vaccine, with the varying components of the microbiota either enhancing or reducing the vaccine’s efficacy. To put an end to the spread of the COVID-19 pandemic, the necessity of vaccines that create powerful and long-term immunity is now more important than ever, and understanding the role of the gut microbiota in this process is essential. Conversely, COVID-19 vaccines also have a significant effect on the gut microbiota, decreasing its total number of organisms and the variety of species present. In this Review, we analyze the evidence that suggesting an interaction between the gut microbiota and COVID-19 vaccine effectiveness, consider the immunological mechanisms that may be responsible for this connection, and explore the possibility of using gut microbiota-focused interventions to improve the efficacy of COVID-19 vaccines.

## Introduction

The impact of the novel coronavirus SARS-CoV-2 on human health has been devastating, resulting in a global pandemic since 2020^1,2^. In 2023, the effects of the COVID-19 pandemic will still be lingering in the world due to the potential for new variants of the virus to emerge and cause further disruption^3^. Rather than hoping for the end, lowering our guard, and thinking that the problems lie elsewhere, the world needs to remain vigilant; encourage maximum transparency in reporting hospital admissions and deaths; and promote collaborative surveillance for testing variants and accelerate vaccination in developing countries^4^. Vaccines are key to ending this pandemic and providing protection to the population. Vaccines work by stimulating the body’s immune system to produce antibodies that bind to and recognize the virus, thereby preventing it from entering cells and causing infection. Vaccines also stimulate the body’s immune system to produce T-cells, which recognize and destroy infected cells^5,6^. However, many factors may influence the immunogenicity, efficacy, and safety of a vaccine. The gut microbiota is a complex and dynamic ecosystem of microorganisms that inhabit the gastrointestinal tract. Composed of bacteria, fungi, viruses, and archaea, it is essential for human health. The gut microbiota plays a role in digestion, metabolism, immunity, and protection against pathogens. It is also involved in the development and maintenance of the immune system, and has been linked to a variety of diseases, including obesity, diabetes, and cancer^7^. Research has shown that the intestinal microbial population is a critical factor in immune response, and thus it has been suggested that gut microbiota could play a pivotal role in the efficacy of COVID-19 vaccines^8^. On the other hand, COVID-19 vaccines have the potential to affect overall health by regulating the diversity of the gut microbiota and modulating the presence of certain bacteria that are associated with certain diseases^9^. Gut microbiota profiling could be a potential tool to identify individuals who may have suboptimal or excessive immune response to COVID-19 vaccines and tailor personalized interventions accordingly. Given the complexity of the human immune system and the potential for different vaccines to have varying effects, extensive research has been conducted to better understand the relationship between the gut microbiota and COVID-19 vaccines. This review will provide a comprehensive overview of the interaction between gut microbiota and COVID-19 vaccines.

## Current COVID-19 vaccine candidates and potential factors affecting vaccine efficacy

The development of effective vaccine for COVID-19 has been a major focus of research and development efforts since the onset of the pandemic. In January 2023, the World Health Organization (WHO) is determined to maintain the momentum for accelerating COVID-19 vaccination in developing countries^10^. Currently, various vaccine candidates have been developed and approved for use by different national regulatory agencies. These include RNA vaccines, protein subunit vaccines, non-replicating viral vector vaccines, inactivated virus vaccines, virus-like particles (VLP) vaccines and DNA vaccines. By February 2023, a total of 50 vaccines had been approved for use in 201 countries, with 11 of them having received Emergency Use Listing (EUL) from the WHO (Table 1) (https://covid19.trackvaccines.org/). At present, the most widely used COVID-19 vaccines are the Pfizer-BioNTech BNT162b2 vaccine and the Moderna Spikevax vaccine, both of which are mRNA vaccines^11^. Recent studies have shown that mRNA vaccines might be a promising alternative to traditional conventional vaccines due to their high potency, efficacy, safety, and capacity for rapid clinical development. Due to the continued viral evolution and high transmission rates of the Omicron variant, it is difficult to generate long-term immunity against SARS-CoV-2 transmission by current vaccination. However, both BNT162b2 and Spikevax have shown significant efficiency in reducing rates of morbidity and mortality from COVID-19. Currently, all the COVID-19 vaccines listed in Table 1 are parenteral vaccines. They are all given by intramuscular injection. So far, there have been some non-parenteral candidate vaccines under development, including nasal sprays, oral liquids, and oral tablets. The advantages of these vaccines are that they can avoid the discomfort of needle-phobic patients and reduce the cost and difficulty of cold chain transportation. However, these non-parenteral vaccines still need to undergo further clinical trials to prove their safety and efficacy.

**Table 1.** A list of 50 COVID-19 vaccines that have been approved for use in 201 countries.

Vaccine Name	Manufacturer	Type of Vaccine	Approved Status	Number of Completed Clinical Trails	Granted EUL by WHO
Zifivax	Anhui Zhifei Longcom	Protein Subunit	Approved in 4 countries	21 trials in 5 countries	No
Noora vaccine	Bagheiat-allah University of Medical Sciences	Protein Subunit	Approved in 1 country	3 trials in 1 country	No
Corbevax	Biological E Limited	Protein Subunit	Approved in 2 countries	7 trials in 1 country	No
Abdala	Center for Genetic Engineering and Biotechnology (CIGB)	Protein Subunit	Approved in 6 countries	5 trials in 1 country	No
Soberana 02	Instituto Finlay de Vacunas Cuba	Protein Subunit	Approved in 4 countries	7 trials in 2 countries	No
Soberana Plus	Instituto Finlay de Vacunas Cuba	Protein Subunit	Approved in 2 countries	5 trials in 1 country	No
V-01	Livzon Mabpharm Inc	Protein Subunit	Approved in 1 country	7 trials in 3 countries	No
MVC-COV1901	Medigen	Protein Subunit	Approved in 4 countries	15 trials in 4 countries	No
Recombinant SARS-CoV-2 Vaccine (CHO Cell)	National Vaccine and Serum Institute	Protein Subunit	Approved in 1 country	3 trials in 2 countries	No
Nuvaxovid	Novavax	Protein Subunit	Approved in 40 countries	22 trials in 14 countries	Yes
IndoVac	PT Bio Farma	Protein Subunit	Approved in 1 country	4 trials in 1 country	No
Razi Cov Pars	Razi Vaccine and Serum Research Institute	Protein Subunit	Approved in 1 country	5 trials in 1 country	No
VidPrevtyn Beta	Sanofi/GSK	Protein Subunit	Approved in 30 countries	3 trials in 2 countries	No
COVOVAX (Novavax formulation)	Serum Institute of India	Protein Subunit	Approved in 6 countries	7 trials in 3 countries	Yes
SKYCovione	SK Bioscience Co Ltd	Protein Subunit	Approved in 1 country	7 trials in 6 countries	No
TAK-019 (Novavax formulation)	Takeda	Protein Subunit	Approved in 1 country	3 trials in 1 country	No
SpikoGen	Vaxine/CinnaGen Co.	Protein Subunit	Approved in 1 country	8 trials in 2 countries	No
Aurora-CoV	Vector State Research Center of Virology and Biotechnology	Protein Subunit	Approved in 1 country	4 trials in 1 country	No
Razi Cov Pars	Razi Vaccine and Serum Research Institute	Protein Subunit	Approved in 1 country	5 trials in 1 country	No
VidPrevtyn Beta	Sanofi/GSK	Protein Subunit	Approved in 30 countries	3 trials in 2 countries	No
COVOVAX (Novavax formulation)	Serum Institute of India	Protein Subunit	Approved in 6 countries	7 trials in 3 countries	No
SKYCovione	SK Bioscience Co Ltd	Protein Subunit	Approved in 1 country	7 trials in 6 countries	No
TAK-019 (Novavax formulation)	Takeda	Protein Subunit	Approved in 1 country	3 trials in 1 country	No
SpikoGen	Vaxine/CinnaGen Co.	Protein Subunit	Approved in 1 country	8 trials in 2 countries	No
Aurora-CoV	Vector State Research Center of Virology and Biotechnology	Protein Subunit	Approved in 1 country	2 trials in 1 country	No
EpiVacCorona	Vector State Research Center of Virology and Biotechnology	Protein Subunit	Approved in 4 countries	4 trials in 1 country	No
GEMCOVAC-19	Gennova Biopharmaceuticals Limited	RNA	Approved in 1 country	2 trials in 1 country	No
Covifenz	Medicago	RNA	Approved in 1 country	6 trials in 6 countries	No
Spikevax	Moderna	RNA	Approved in 88 countries	70 trials in 24 countries	Yes
Spikevax Bivalent Original/Omicron BA.1	Moderna	RNA	Approved in 38 countries	5 trials in 4 countries	No
Spikevax BivalentOriginal/Omicron BA.4/BA.5	Moderna	RNA	Approved in 33 countries	2 trials in 1 country	No
Comirnaty (BNT162b2)	Pfizer/BioNTech	RNA	Approved in 149 countries	100 trials in 31 countries	Yes
Comirnaty Bivalent Original/Omicron BA.1	Pfizer/BioNTech	RNA	Approved in 35 countries	3 trials in 5 countries	No
Comirnaty Bivalent Original/Omicron BA.4/BA.5	Pfizer/BioNTech	RNA	Approved in 33 countries	4 trials in 1 country	No
TAK-919 (Moderna formulation)	Takeda	RNA	Approved in 1 country	2 trials in 1 country	No
AWcorna	Walvax	RNA	Approved in 1 country	6 trials in 1 country	No
Covaxin	Bharat Biotech	Inactivated Virus	Approved in 14 countries	16 trials in 2 countries	Yes
KoviVac	Chumakov Center	Inactivated Virus	Approved in 3 countries	3 trials in 1 country (No trials for Phase 3)	No
Turkovac	Health Institutes of Turkey	Inactivated Virus	Approved in 1 country	8 trials in 1 country	No
FAKHRAVAC (MIVAC)	Organization of Defensive Innovation and Research	Inactivated Virus	Approved in 1 country	3 trials in 1 country	No
QazVac	Research Institute for Biological Safety Problems (RIBSP)	Inactivated Virus	Approved in 2 countries	3 trials in 1 country	No
KCONVAC	Shenzhen Kangtai Biological Products Co	Inactivated Virus	Approved in 2 countries	7 trials in 1 country	No
COVIran Barekat	Shifa Pharmed Industrial Co	Inactivated Virus	Approved in 1 country	6 trials in 1 country	No
Covilo	Sinopharm (Beijing)	Inactivated Virus	Approved in 93 countries	39 trials in 18 countries	Yes
Inactivated (Vero Cells)	Sinopharm (Wuhan)	Inactivated Virus	Approved in 2 countries	9 trials in 7 countries	No
CoronaVac	Sinovac	Inactivated Virus	Approved in 56 countries	42 trials in 10 countries	Yes
VLA2001	Valneva	Inactivated Virus	Approved in 33 countries	9 trials in 4 countries	No
iNCOVACC	Bharat Biotech	Non-Replicating Viral Vector	Approved in 1 country	4 trials in 1 country	No
Convidecia	CanSino	Non-Replicating Viral Vector	Approved in 10 countries	14 trials in 6 countries	Yes
Convidecia Air	CanSino	Non-Replicating Viral Vector	Approved in 2 countries	5 trials in 4 countries	No
Gam-COVID-Vac	Gamaleya	Non-Replicating Viral Vector	Approved in 1 country	2 trials in 1 country (No trials for Phase 3)	No
Sputnik Light	Gamaleya	Non-Replicating Viral Vector	Approved in 26 countries	7 trials in 3 countries	No
Sputnik V	Gamaleya	Non-Replicating Viral Vector	Approved in 74 countries	25 trials in 8 countries	No
Jcovden	Janssen (Johnson & Johnson)	Non-Replicating Viral Vector	Approved in 113 countries	26 trials in 25 countries	Yes
Vaxzevria	Oxford/AstraZeneca	Non-Replicating Viral Vector	Approved in 149 countries	73 trials in 34 countries	Yes
Covishield (Oxford/AstraZeneca formulation)	Serum Institute of India	Non-Replicating Viral Vector	Approved in 49 countries	6 trials in 1 country	Yes
Covifenz	Medicago	Virus-like Particles (VLP)	Approved in 1 country	6 trials in 6 countries	No
ZyCoV-D	Zydus Cadila	DNA	Approved in 1 country	6 trials in 1 country	No

**Figure 1.**
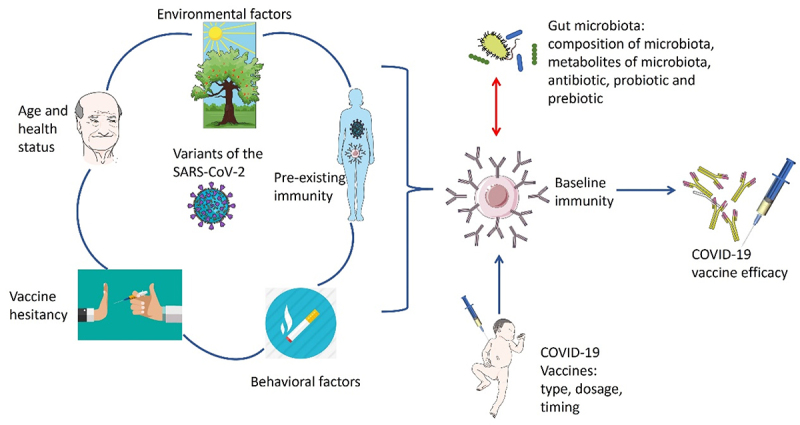
Potential factors influencing the efficacy of the COVID-19 vaccine.

It is evident that there is a pressing need for COVID-19 vaccines that are highly effective and can generate strong and long-lasting immunity. However, why immune system responses to current COVID-19 vaccination vary a lot between different individuals and populations is still not fully understood. There are several factors that may potentially influence the current efficacy of COVID-19 vaccines (Figure 1), including the following.

Various host factors and vaccine-related factors may contribute to the body’s response to the COVID-19 vaccine, such as the variants of the virus, age and health status, vaccine type, dosage, and timing; preexisting immunity, vaccine hesitancy, environmental and behavioral factors.

### Variants of the virus

Emerging variants of the SARS-CoV-2 virus, such as the current Omicron variant, have shown to be less susceptible to vaccine-induced immunity^12^. There is concern about the ability of the current vaccines to protect against emerging viral variants. Mutations in the S-glycoprotein may affect transmission dynamics and the risk of immune escape. However, full vaccination of COVID-19 vaccines is highly effective against Alpha variant, and moderate effective against Beta, Gamma, and Delta variants^13^. Booster vaccination is more effective against Delta and Omicron variants. mRNA vaccines seem to have higher vaccine efficacy against Alpha, Beta, Gamma, and Delta variants over others. Many studies have shown that the efficacy of vaccines against Omicron variant is lower than against previous variants of the SARS-CoV-2 virus^14^.

### Age, sex, health and nutritional status

The efficacy of COVID-19 vaccines can be influenced by an individual’s age, sex, health and nutritional status in several ways: (1) Age: Older adults are at a higher risk for severe illness and death from COVID-19, and they may have weaker immune systems, which can reduce the efficacy of the vaccine. Some studies have found that older adults have a slightly lower immune response to the vaccine compared to younger people^15^. (2) Sex: Sex differences may also exist in COVID-19 vaccine efficacy, as women may have stronger immune responses and more adverse reactions than men, due to hormonal and genetic factors^16^ (3) Underlying health conditions: People with underlying health conditions such as diabetes, cancer, heart disease, and obesity are more likely to experience severe illness from COVID-19. They may also have a weaker immune response to the vaccine, which can reduce its efficacy^17^. (4) Immune status: People with weakened immune systems, such as the HIV-infected people or patients who are taking immunosuppressive medications, may not have a strong immune response to the COVID-19 vaccine, which can reduce its efficacy^18^. (5) Nutritional status: Nutritional status may affect COVID-19 vaccine efficacy by modulating the immune system and influencing inflammation and oxidative stress^19^.

### Vaccine type, dosage, timing and administration route

The efficacy of COVID-19 vaccines may be influenced by the type, dosage, and timing of vaccination. Different types of COVID-19 vaccines have varying levels of efficacy, and some may be more effective in certain populations than others. For example, the mRNA vaccines from Moderna and Pfizer-BioNTech have shown potential higher efficacy rates in clinical trials compared to other vaccines^17^. The dosage of a vaccine refers to the amount of the vaccine antigen that is included in each dose. A higher dosage may produce a stronger immune response, but it may also increase the risk of side effects. Conversely, a lower dosage may produce a weaker immune response, but it may also reduce the risk of side effects. The timing of COVID-19 vaccination can also influence the efficacy of the vaccine, particularly in relation to the emergence of novel variants of the SARS-CoV-2 virus. Studies have shown that the timing of the booster dose can impact the level of protection against new variants^20^. At least six months after receiving their second dose of the Pfizer-BioNTech vaccine, it has been found that a third dose provides significantly increased protection against the Omicron variant. Similarly, a study published in 2021 found that the efficacy of the AstraZeneca vaccine against the Delta variant was significantly higher when the second dose was given at a 12-week interval rather than a 6-week interval^13^. The administration route of the vaccine may affect its efficacy by influencing the type and magnitude of immune response elicited. For example, intramuscular injection may induce higher levels of systemic antibodies, while intranasal or oral delivery may induce higher levels of mucosal immunity^21^.

### Pre-existing immunity

Pre-existing immunity can have a significant impact on the efficacy of the COVID-19 vaccine. Natural immunity from a previous SARS-CoV-2 infection can enhance the efficacy of vaccine by providing an additional layer of protection against the virus. Studies have shown that individuals who have had a prior COVID-19 infection possessed elevated T-cell reactions and concentrations of neutralizing antibodies after vaccination than those who have not been infected before. A study published in 2021 looked at the immune response of healthcare workers in the UK who had either been previously infected with SARS-CoV-2 or had no prior infection^22^. Another study investigated the immune response of individuals who received the Pfizer-BioNTech vaccine and had a previous COVID-19 infection. The study found that these individuals had elevated T-cell reactions and higher concentrations of neutralizing antibodies than those who had received the vaccine but had no prior infection^23^. Above studies suggest that natural immunity from a prior COVID-19 infection can enhance the efficacy of the vaccine by boosting the immune response to the virus. However, more research is required to fully understand the extent of this effect and how long it lasts. It is still recommended that individuals receive the COVID-19 vaccine even if they have had a previous infection.

### Vaccine hesitancy

Vaccine hesitancy, or the reluctance or refusal to get vaccinated, can have a significant impact on the efficacy of the COVID-19 vaccine. When a large percentage of the population is not vaccinated, the virus can continue to circulate and mutate, potentially leading to new variants that may be more transmissible, virulent, or resistant to current vaccines. Additionally, unvaccinated individuals may be at higher risk of contracting and spreading the virus, potentially overwhelming healthcare systems and prolonging the pandemic^24^. Several studies suggest that vaccine hesitancy has contributed to lower vaccination rates in some populations, which can ultimately impact the effectiveness of the COVID-19 vaccine. For example, a study by Moore et al. found that vaccine hesitancy was more common among certain groups, including Black, Hispanic, and rural populations in the United States^25^. Generally, vaccine hesitancy can have an obvious impact on the effectiveness of the COVID-19 vaccine by contributing to lower vaccination rates and potentially allowing the virus to continue to circulate and mutate. Efforts to address vaccine hesitancy through education and communication campaigns may be crucial in improving vaccination rates and ensuring the effectiveness of the COVID-19 vaccines.

### Environmental factors

Environmental factors can influence the efficacy of the COVID-19 vaccines in several ways. These include factors such as temperature and storage conditions, prevalence of the virus in the local community as well as the air pollution^26^. It is important to properly store and transport the vaccine, and efforts to reduce air pollution and virus transmission rates may also help to improve the efficacy of the vaccine.

### Behavioral factors

Several studies have explored how specific behavioral factors such as smoking, exercise, and physiological stress may influence the efficacy of the COVID-19 vaccine, but more research is needed to fully understand the extent of these effects^24^. Encouraging healthy behaviors such as regular exercise and smoking cessation, as well as addressing sources of physiological stress, may help to optimize the immune response to the COVID-19 vaccine.

Above studies have indicated that various host factors and vaccine-related factors may contribute to the body’s response to the COVID-19 vaccine. Furthermore, increasing evidence has demonstrated that intestinal microbes may be a critical and modifiable factor that could influence the immune system and the reaction to the COVID-19 vaccine. It is noteworthy that several factors may influence the estimated efficacy of COVID-19 vaccines in non-randomized studies, such as the study design, population characteristics, vaccine type and dose, outcome definition and ascertainment, exposure and outcome measurement, confounding variables, and statistical methods. These factors may introduce heterogeneity, bias, and uncertainty in the estimates of vaccine efficacy, limiting their generalizability and comparability across different settings and populations. Therefore, it is important to assess the quality and validity of non-randomized studies using appropriate tools and criteria, such as the Newcastle-Ottawa Scale or the ROBINS-I tool^27,28^. Additionally, it is essential to report the details of the study methods and results transparently and comprehensively, following the STROBE guidelines or other relevant reporting standards^29^. Non-randomized studies can provide valuable information on the real-world effectiveness of COVID-19 vaccines, but they should be interpreted with caution and supplemented by data from randomized controlled trials whenever possible.

## The mutual effects between COVID-19 vaccines and gut microbiota

It is estimated that trillions of bacteria inhabit our intestines, which is 10 times more than the number of eukaryotic cells in our bodies. Recent studies have indicated that this microbial population, known as the ‘second genome’, has a significant influence on our physiology, affecting our intestinal immunity, metabolism, allergic inflammation and autoimmune, and even our communication with the central nervous system^12,30^. Furthermore, changes in microbiome composition have been associated with modulation of the efficacy of various immune interventions, including COVID-19 vaccination^30,31^. While the gut microbiota can influence the immunogenicity and efficacy of COVID-19 vaccines, the reverse is also true: COVID-19 vaccines can have significant effects on the gut microbiota, reducing the total number of organisms and diversity of species. This bidirectional interaction between gut microbiota and COVID-19 vaccines is highly reminiscent of that between gut microbiota and anticancer immunotherapies.

The interaction between gut microbiota and COVID-19 vaccines has been the subject of intense research in recent months. While the development of effective vaccines for the virus has been a major step forward in combating the pandemic, there is still much to learn about how the gut microbiota will affect vaccine efficacy. It is well established that the intestinal microbial population plays a pivotal role in the immune response, and it can play a role in both the efficacy and safety of COVID-19 vaccines. Additionally, the COVID-19 vaccines have also been shown to modulate the diversity of the gut microbiota, which can lead to an imbalance in the gut microbiome^32^. In the following parts, we evaluate the data from animal experiments, clinical interventional studies, and observational studies to demonstrate the mutual effects between COVID-19 vaccines and gut microbiota.

### Animal experiments

Several *in vivo* research have reported that the intestinal microbial population may influence the immune response to COVID-19 vaccines in animal models. A study conducted recently investigated the origin of antibodies that were already present and reacted to SARS-CoV-2, as well as their possible effects on the efficacy of a vaccine^33^. The SARS-CoV-2 spike protein consists of two subunits: S1 and S2. The S1 subunit contains the receptor-binding domain (RBD) that binds to the host cell receptor angiotensin-converting enzyme 2 (ACE2). The S2 subunit mediates the fusion of the viral and host cell membranes. It was found that the S2 subunit was the main target of these antibodies in mice model. The S2 subunit contains a connector domain (CD) that links the RBD to the fusion peptide (FP), a heptad repeat 1 (HR1) region that forms a trimeric coiled-coil structure, a heptad repeat 2 (HR2) region that interacts with HR1 to form a six-helix bundle (6-HB), and a transmembrane domain that anchors the spike protein to the viral envelope. The connector domain of S2 was found to have a dominant antibody epitope, which was already recognized by antibodies present in mice. Evidence from metagenomic sequencing and fecal bacteria transplants showed that the production of S2 cross-reactive antibody is related to commensal intestinal microbes. Additionally, 6 monoclonal antibodies that reacted with P144 were obtained from mice and commensal intestinal bacteria can cross-react with the antibodies. P144 is a synthetic peptide derived from the CD region of the S2 subunit, which has been shown to inhibit SARS-CoV-2 infection by interfering with the conformational changes of the spike protein required for membrane fusion. This study uncovered an alternate source of preexisting antibodies that target the S2 protein, which were observed to have increased levels in mice after they were administered a SARS-CoV-2 S DNA vaccine. Furthermore, it also shed light on the influence that gut microbiota can have on a host’s immunity to SARS-CoV-2, which has until now been largely overlooked. Another study by Geanes et al. described the design of a novel peptide-conjugate SARS-CoV-2 vaccine containing conserved S2 spike epitope with mice model^34^. After immunizing the mice, it was found that the epitope elicited cross-reactive antibodies could bind with various coronaviruses. Interestingly, the shared sequence homology between the epitope of the vaccine and proteins in commensal intestinal microbiota led to changes in the microbiota, which in turn affected the immune response to the vaccine. This caused a decrease in the vaccine’s efficacy and reduced its ability to protect against SARS-CoV-2 infection. A recent study by Xu et al. has demonstrated that the probiotic strain *Lactobacillus* plantarum GUANKE (LPG) might boost and prolong COVID-19 specific immunological reactions in both effective and memory phases in mice model^35^. Giving LPG orally was found to increase neutralizing antibodies against SARS-CoV-2, even 6 months after immunization. When the vaccine and LPG were administered at the same time, the neutralization antibodies were enhanced and T-cell responses were lasting and steady for an extended period. Gene expression tests demonstrated that taking LPG orally triggered immune reactions in mucosal and systemic compartments. These findings imply that probiotics may be used in conjunction with SARS-CoV-2 vaccines to raise their potency. While the results from these animal studies are promising, further research is required to exam whether similar effects occur in humans. Nevertheless, these findings from animal studies highlight the potential importance of gut microbiota in COVID-19 vaccines development and could have significant implications for improving vaccine efficacy and safety in the future.

### Clinical interventional studies

Several clinical interventional studies have investigated the role of gut microbiota in influencing the immune response to COVID-19 vaccines. Most of these studies have focused on the application of probiotics, while some have also examined the potential roles of prebiotics and antibiotics in COVID-19 vaccination. Recently, a trial tested how the probiotic *Loigolactobacillus* coryniformis K8 (LCK8) affects healthcare workers (HCWs) who face a high risk of infection from COVID-19.^36^. The trial was randomized, double-blind, and placebo controlled. It measured how COVID-19 infection and severity, immunological reactions, and vaccine side effects changed after using probiotics. 250 frontline HCWs over the age of 20 took either a placebo or LCK8 every day for 2 months. The sub-cohort consisted of 95 subjects who had COVID-19 during the intervention period, of whom 47 received LCK8 and 48 received placebo. In this trail, the probiotic group orally received 2 × 10^9^ CFU of LCK8 in a capsule form once daily. The placebo group received a capsule containing maltodextrin and lactose. The capsules were identical in appearance and taste. An antigen or PCR test was applied for examining COVID-19 infection. The volunteers who got vaccinated during the trial had their specific IgG in serum checked at the end of the trial. Generally, COVID-19 infection was rare, and there was no big difference between the groups. The sample was divided according to the days between the first dose and antibody test for the purpose of assessing immune response. Over time, the specific IgG levels decreased. However, in the subset of subjects who had more than 81 days between their first dose and the antibody test, those who took LCK8 had notably higher specific IgG levels than those in the control group. Also, those who began consuming probiotics prior to receiving their initial vaccine dose had substantially less side effects, especially soreness in their arm, compared to those who did not. To sum up, the probiotic LCK8 helps high-risk populations maintain their immune protection from the COVID-19 vaccine for longer.

A recent study by Wong et al. investigated the effects of a novel oral microbiome formula (SIM01) on reducing adverse health outcomes among elderly and diabetes patients who were naive to COVID-19 vaccination during the pandemic^37^. This study was a single-center, double-blind, randomized, placebo-controlled trial involving 453 volunteers who received either SIM01 or a placebo (vitamin C) for three months within one week of the first dose of either the mRNA vaccine BNT162b2 or the inactivated vaccine Sinovac-CoronaVac. The study showed that the SIM01 group had significantly lower rates of adverse health outcomes, such as respiratory infections, gastrointestinal symptoms, and skin problems, than the placebo group. The study also showed that the SIM01 group had better sleep quality, skin condition, and mood than the placebo group. The study also showed that the SIM01 group had a significant increase in beneficial *Bifidobacteria* and butyrate-producing bacteria in fecal samples, strengthened the microbial ecology network and the potential for improving COVID-19 vaccine efficacy.

A recent study investigated the effects of a probiotic-based nutritional supplement in people who had received the influenza or COVID-19 vaccination^38^. This study was a randomized, single-center, placebo-controlled, double-blind trial involving 72 volunteers. Subjects in the study were randomly given either ABBC1®, a supplement containing a probiotic complex, or a placebo on the day after receiving either the influenza or COVID-19 vaccine. Treatment duration was 28 days for the influenza vaccine group and 35 days for the COVID-19 vaccine group. In this study, the ABBC1® group orally received a sachet containing 5 × 10^9^ CFU of a probiotic complex (*Lactobacillus acidophilus* LA02, *Lactobacillus plantarum* LP01, *Bifidobacterium breve* BR03, and *Bifidobacterium lactis* BS01) plus prebiotics (fructooligosaccharides and inulin) once daily. The placebo group received a sachet containing maltodextrin and lactose. In the COVID-19 group, CD4+T cells increased from 910 to 1000 cells/mL in the ABB C1® group, while they decreased from 1054 to 928 cells/mL in the placebo group. CD8+T and CD3+T lymphocytes had a similar trend. Serum levels of IgM and IgG increased more in the ABBC1® group than in the placebo group. No obvious adverse effects or tolerance issues related to ABBC1® were found. These results suggest that ABBC1® could improve the efficacy of both the influenza and COVID-19 vaccines. This effect might be related to the modulation of gut microbiota dysbiosis by ABBC1®.

An study conducted by the University of Hong Kong investigated whether recent antibiotic use could led to gut dysbiosis and affect the immunogenicity of the BNT162b2 vaccine^39^. A total of 310 BNT162b2 vaccine recipients were recruited from three centers and their seroconversion of neutralizing antibody (NAb) was measured at 21, 56 and 180 days post the first dose. Out of these, 29 (9.2%) had taken antibiotics within six months of being vaccinated. Decreased seroconversion rates were observed in antibiotic treatment group at day 21 and day 56, although no such difference was seen at day 180. After adjusting for other variables, recent antibiotic treatment was found to be related with decreased seroconversion rate at day 21. Additional factors related with decreased seroconversion after the first dose of BNT162b2 included age ≥60 years and male sex. However, no significant factors were associated with seroconversion after two doses of BNT16b2, including antibiotic treatment. These results indicate that recent antibiotic use may be connected to a lower seroconversion rate at day 21 among BNT162b2 recipients. Further research with a larger sample size is needed to evaluate the impact of antibiotics on immunogenicity and the durability of vaccination reactions.

A recent clinical interventional study has been conducted in Australia since October 2021, investigating the potential of dietary inulin supplementation to improve vaccine responsiveness in kidney transplant recipients (KTRs) who are at a high risk of hospitalization and death from COVID-19^31^. This randomized, multicenter, placebo-controlled, prospective, double-blinded pilot trial seeks to investigate the consequences of consuming inulin before administering the third dose of the COVID-19 vaccine to patients who have undergone kidney transplants and have not developed immunity following two doses vaccine. The researchers hypothesized that inulin may regulate gut microbiota dysbiosis and promote vaccine responsiveness in KTRs. Participants will be randomly assigned 1:1 to receive either inulin or placebo for four weeks before and after vaccination. In this trial, the inulin group will orally receive 20 g/day of inulin powder (Orafti® P95) dissolved in water or juice. The placebo group will receive 20 g/day of maltodextrin powder (Cargill®) dissolved in water or juice for the same duration. This study seeks to determine the proportion of participants in each trial who develop in vitro neutralization of live SARS-CoV-2 virus 28 days after receiving a third dose of COVID-19 vaccine. Additionally, it will investigate the safety and tolerability of dietary inulin, the diversity and differential abundance of gut microbiota, and vaccine-specific immune cell populations and responses. The inulin for 3rd-dose vaccination stimulation trial will attempt to determine if dietary interventions that alter the gut microbiota can be used to improve vaccine efficacy. So far, this study has not been completed and no results have been released. However, this study might provide a potential accessible, cost-effective, and scalable way to enhance COVID-19 vaccine responses in the future. The heterogeneity of probiotic strains, types of prebiotics, doses, administration modes, and study populations presents a challenge in evaluating the effects of probiotics and prebiotics on COVID-19 vaccine responsiveness. Different type of probiotics and prebiotics may have distinct mechanisms of action and interactions with the host immune system and gut microbiota, and the dose and administration mode of probiotics and prebiotics may affect their viability, colonization, and activity in the gut^40^. Furthermore, the study population may influence the response to probiotics, prebiotics, and COVID-19 vaccines, as factors such as age, health status, diet, medication use, and microbiota composition may vary among individuals and groups. Consequently, it is difficult to compare and generalize the results of different studies and to determine the optimal type, dose, and duration of probiotics and prebiotics intervention for enhancing COVID-19 vaccine efficacy and safety^41^. Therefore, more standardized and well-designed studies are needed to address these issues and to provide evidence-based recommendations for the use of probiotics or other interventions in conjunction with COVID-19 vaccines.

Overall, the studies above suggests that the intestinal microbiota may play a pivotal role in modulating vaccine efficacy and safety. Further research is needed to confirm and expand on these findings and to identify the optimal gut microbiota modulation strategies to enhance vaccine response. Nonetheless, these clinical interventional studies provide evidence that the intestinal microbiota may affect the efficacy and safety of COVID-19 vaccines.

### Clinical observational studies

In a prospective, clinical observational cohort study of 42 infliximab-treated patients with inflammatory bowel disease (IBD) undergoing vaccination against SARS-CoV-2, researchers examined whether the gut microbiota and metabolome could account for the variation in the efficacy of the COVID-19 vaccines^32^. Of the 43 patients studied, 30 had Crohn’s disease, 13 had ulcerative colitis, and 1 had IBD-unclassified; 26 of the patients were taking thiopurine therapy. 8 of the patients had already been exposed to SARS-CoV-2. Those with below average vaccination responses had lower gut microbiota diversity. Having more *Bilophila* in the gut was linked to a better serological response, whereas having more *Streptococcus* was linked to a poorer response. The fecal metabolome of those with above and below average responses was also distinct. Omega-muricholic acid, trimethylamine, and isobutyrate were connected to a better response, while phenylalanine, succinate, taurodeoxycholate, and taurolithocholate were linked to a poorer response. These findings indicate that there is a connection between the intestinal microbes and the varying levels of protection offered by the COVID-19 vaccine in immunocompromised patients. Bacterial metabolites such as trimethylamine may play a role in countering the immunosuppressive effects of anti-TNF therapy.

In a recent prospective, observational study, Ng et al. examined the gut microbiota composition in 138 adults who had received either the inactivated CoronaVac or the mRNA BNT162b2 vaccine^42^. The researchers used shotgun metagenomic sequencing in stool samples collected at baseline and one month after the second dose of vaccine. The SARS-CoV-2 surrogate virus neutralization test and the spike receptor-binding domain IgG ELISA were used to measure immune markers. Their results found that recipients of CoronaVac showed a remarkable decreased immunological reaction than BNT162b2 vaccinees. Subjects with high neutralizing antibodies to CoronaVac vaccine had a persistent presence of *Bifidobacterium* adolescentis. The abundance of *Prevotella* copri and two *Megamonas* species were found to be higher in individuals with fewer adverse events following either of the BNT162b2 vaccines, suggesting that these bacteria may have an anti-inflammatory role in the host immune response. Additionally, the baseline gut microbiome of individuals who received the BNT162b2 vaccine was found to be enriched in pathways associated with carbohydrate metabolism. Furthermore, a positive correlation was observed between the number of neutralizing antibodies and the abundance of bacteria with flagella and fimbriae, such as *Roseburia faecis*. Furthermore, it was discovered that individuals with fewer adverse effects after receiving either of the vaccines had higher levels of *Prevotella* copri and two *Megamonas* species, indicating that these bacterial species may have an anti-inflammatory role in the host immune system. These findings suggest that particular gut microbiota markers are associated with improved vaccine efficacy and reduced vaccine-related adverse events in people who have received COVID-19 vaccines, implying that microbiota-targeted interventions could be beneficial as an adjunct to enhance the effectiveness of the vaccine.

An clinical observational study conducted in Singapore investigated the effects of BBIBP-CorV, an inactivated SARS-CoV-2 vaccine candidate, on the gut microbiota of healthy adults^30^. The researchers compared the microbial composition before and after vaccination and found that microbial diversity, as measured by the Simpson, Shannon, Pielou Evenness and Invsimpson indices, was significantly reduced. *Ruminococcus* and Actinomyces were notably decreased, while *Faecalibacterium* was significantly increased. The findings of the study suggest that the BBIBP-CorV vaccine may be able to modulate the composition and functions of the gut microbiome, which could lead to protection from COVID-19. Specifically, the potential functional profiles of the gut microbiome related to amino acid metabolism, lipid biosynthesis proteins, and steroid biosynthesis were significantly increased, while the capacity for the renin-angiotensin system was significantly reduced.The COVID-19 pandemic also increased the rates of anxiety and depression. To treat these problems effectively, a study conducted by a Spanish group looked at how gut microbes relate to mental health such as anxiety, depression, or post-traumatic stress disorder (PTSD), after the COVID-19 infection or BNT162b2 vaccination^43^. They analyzed gut microbes by 16S ribosomal RNA gene V3–4 amplicon sequencing from stool samples of 198 people who answered questionnaires about their mental health. They looked at gut microbial diversity and structure, and how much of each type of microbe was present. In this study, 17% had depression symptoms, 41% had trait anxiety symptoms,37% had state anxiety symptoms, and 8% had PTSD symptoms. Many people had more than one problem. People with trait anxiety had less diverse gut microbes. People with depression + PTSD + state and trait anxiety symptoms had less *Fusicatenibacter* saccharivorans, while people with depression symptoms had more *Proteobacteria* and less *Synergistetes* phyla. The amount of *Anaerostipes* was linked to childhood trauma, and people who faced life-threatening traumas had more *Turicibacter* sanguinis and less *Lentisphaerae*. COVID-19 infection and BNT162b2 vaccination significantly changed the overall gut microbial makeup and were linked to different amounts of each type of microbe. These findings indicated that gut microbes might affect anxiety, depression, and PTSD symptoms and COVID-19 vaccination might improve mental health by modulating gut microbial makeup.

A group in China also investigated whether gut microbes could affect COVID-19 vaccines work^44^. They studied how gut microbes and their activities related to the BBIBP-CorV vaccination reactions. This study included 200 people who have got BBIBP-CorV vaccination. The researchers use metagenomic sequencing and metabolomic assays to study their intestinal microbes and their activities. The results showed that the BBIBP-CorV vaccine changed the composition of gut microbes and related functional pathways. The high antibody response group has more short-chain fatty acids (SCFAs) than the low response group, and some SCFAs are linked to a better antibody response. This study showed that gut microbes and their activities are associated with the BBIBP-CorV vaccine response, providing us with clues as to how to modulate gut microbes to improve the efficiency of current COVID-19 vaccines.

Another group in China assessed the host’s response after vaccination and investigated the role of gut microbiota in antibodies production^7^. The researchers studied how the Sinovac vaccine affects the blood and gut microbiota of 30 young volunteers (15 men and 15 women, aged 20–23). They collected 143 fecal and 120 blood samples at different times and used various methods to measure their blood immunity and gut microbiota composition. They also compared these results with a published dataset of COVID-19 patients’ gut microbiota. Their results found that vaccination did not change the gut microbiota as much as SARS-CoV-2 infection did. Furthermore, they found that gut microbiota, cytokines, lymphocytes, and SARS-CoV-2 antibodies are related. Some gut microbes are linked to higher or lower levels of IgG. For example, *Prevotella* copri lowers IgG, while *Clostridium* leptum, *Lactobacillus* ruminis, *Ruminococcus* torques and others raise it (all *p* < 0.01). This study also found that gut microbiota and body features have the most significant effect on antibody production. This implies that gut microbiota is essential for generating SARS-CoV-2 antibodies in young, healthy individuals. However, this study has not explored how this works for older people, and further research is needed in this area.

Several studies have reported changes in gut microbiota composition and function following COVID-19 vaccination. For example, Ng et al. demonstrated that both the inactivated vaccine (CoronaVac; Sinovac) and the mRNA vaccine (BNT162b2; BioNTech) altered the gut microbiota composition in 138 healthy adults who received either vaccine^42^. The study found that both vaccines reduced the overall abundance and diversity of gut microbes, as well as the levels of short-chain fatty acids (SCFAs), which are important metabolites for immune regulation and intestinal health. Additionally, the study revealed that certain taxa, such as *Bifidobacterium adolescentis, Faecalibacterium prausnitzii*, and *Ruminococcus bromii*, were associated with higher or lower vaccine immunogenicity and adverse events. Data published online by the Centers for Disease Control and Prevention of the United States (https://www.cdc.gov/coronavirus/2019-ncov/) indicate that there is no significant difference in mild diarrhea between COVID-19 vaccine and placebo recipients. However, the bottom row of data for both first and second injections appears to indicate a signal of increased severe diarrhea among vaccine recipients, suggesting that the diversity of gut microbiota may be reduced, resulting in a 3.2-fold increase in severe diarrhea for the first injection and a 3.8-fold increase for the second injection. The mechanisms by which COVID-19 vaccines affect the gut microbiota are not yet fully understood, but some possible explanations have been proposed. One possibility is that the vaccines induce systemic immune responses that alter the intestinal immune environment and thus affect the growth and survival of gut microbes^45^. Another possibility is that the vaccines induce local immune responses in the gut-associated lymphoid tissue (GALT), which is the largest immune organ in the body and contains many antigen-presenting cells and lymphocytes that can recognize and respond to vaccine antigens^46^. Furthermore, the vaccines may affect the expression and function of ACE2 receptors in the intestinal epithelium, which are not only involved in SARS-CoV-2 entry, but also in maintaining intestinal homeostasis and balancing the microbiota^45^.

In addition to the above-published clinical studies, there are three clinical trials that have yet to be published that are being conducted on ClinicalTrials.gov to examine the interaction between COVID-19 vaccines and gut microbiota by March 2023 (Table 2).

**Table 2.** Summarizes of the ongoing clinical trials testing the interaction between gut microbiota and COVID-19 vaccines.

Study title	Study type	Locations	Status	Study details	Results	Reference
Evaluation of the effect of Loigolactobacillus coryniformis K8 CECT 5711 consumption in health care workers exposed to COVID-19	Interventional study	Spain	Completed	The study population consisted of health care workers exposed to COVID-19 who were aged 18 years or older and had no history of COVID-19 infection or vaccination. The control group received a placebo capsule identical in appearance and taste to the intervention capsule for 12 weeks.	The probiotic L. coryniformis K8 CECT 5711 helps HCWs maintain their immune protection from the COVID-19 vaccine for longer.	^36^
Effect and Tolerability of a Nutritional Supplement Based on a Synergistic Combination of beta-Glucans and Selenium- and Zinc-Enriched Saccharomyces cerevisiae (ABB C1((R))) in Volunteers Receiving the Influenza or the COVID-19 Vaccine: A Randomized, Double-Blind, Placebo-Controlled Study	Interventional study	Spain	Completed	The study population consisted of healthy volunteers aged 18 to 65 years who received either the influenza vaccine (Chiromas®) or the COVID-19 vaccine (Comirnaty®). The control group received a placebo supplement identical in appearance and taste to the intervention supplement for the same duration as the intervention group.	The probiotic-based nutritional supplement ABB C1® could improve the efficiency of both the influenza and COVID-19 vaccines. This effect might relate with the modulation of gut microbiota dysbiosis by ABB C1®.	^38^
Association between Recent Usage of Antibiotics and Immunogenicity within Six Months after COVID-19 Vaccination	Interventional study	China	Completed	The study population consisted of 316 healthy volunteers aged 18 to 65 years who received the BNT162b2 vaccine against COVID-19. The control group consisted of 287 volunteers who had not used any antibiotics within six months before the first dose of the vaccine.	Recent antibiotic use may be associated with a lower seroconversion rate at day 21 (but not day 56 or 180) among BNT162b2 recipients.	^39^
Rapamycin and inulin for third-dose vaccine response stimulation (RIVASTIM): Inulin – study protocol for a pilot, multicentre, randomized, double-blinded, controlled trial of dietary inulin to improve SARS-CoV-2 vaccine response in kidney transplant recipients	Interventional study	Australia	Uncompleted	The study population consists of kidney transplant recipients who have received two doses of COVID-19 vaccine but have not developed protective immunity, defined as a neutralizing antibody titer of less than 30%. The control group receives a daily dose of maltodextrin (20 g) as a placebo for the same duration as the intervention group.	Not available	^31^
The gut microbiota and metabolome are associated with diminished COVID-19 vaccine-induced antibody responses in immunosuppressed inflammatory bowel disease patients	Observational study	United Kingdom	Completed	The study population consists of patients with inflammatory bowel disease (IBD) who are treated with infliximab and have received two doses of COVID-19 vaccine (either ChAdOx1 nCoV-19 or BNT162b2). The control group consists of patients who have a serological response below the geometric mean of the wider CLARITY-IBD cohort, defined as a neutralizing antibody titer of less than 30%.	there is an association between the gut microbiota and variable serological response to vaccination against SARS-CoV-2 in immunocompromised patients. Microbial metabolites including trimethylamine may be important in mitigating anti-TNF-induced attenuation of the immune response.	^32^
Gut microbiota composition is associated with SARS-CoV-2 vaccine immunogenicity and adverse events.	Observational study	China	Completed	The study population consists of adults who have received either the inactivated vaccine (CoronaVac; Sinovac) or the mRNA vaccine (BNT162b2; BioNTech; Comirnaty) against SARS-CoV-2. The control group consists of patients who have a gut microbiota composition that is associated with poorer immune response and more adverse events to COVID-19 vaccination.	specific gut microbiota markers are associated with improved immune response and reduced adverse events following COVID-19 vaccines, suggesting that microbiota-targeted interventions may complement the effectiveness of COVID-19 vaccines.	^42^
Characterization of the Intestinal Microbiome in Healthy Adults over Sars-Cov-2 Vaccination	Observational study	Singapore	Completed	The study population consists of healthy adults who have received two doses of inactivated SARS-CoV-2 vaccine (BBIBP-CorV). The control group consists of patients who have a gut microbiota and metabolome profile that is associated with poorer serological response to COVID-19 vaccination.	The BBIBP-CorV vaccine could modulate the gut microbial composition and functions, which may help to protect against COVID-19.	^30^
The gut-microbiota-brain axis in a Spanish population in the aftermath of the COVID-19 pandemic: microbiota composition linked to anxiety, trauma, and depression profiles.	Observational study	Spain	Completed	The study population consists of adults from Spain who have experienced symptoms of anxiety, depression, or PTSD in the aftermath of the COVID-19 pandemic and/or previous traumatic events. The control group consists of patients who have a gut microbiota composition that is associated with higher levels of anxiety, depression, or PTSD symptoms.	gut microbes might affect anxiety, depression, and PTSD symptoms and COVID-19 vaccination might improve mental health by modulating gut microbial makeup.	^43^
Correlation of gut microbiota and metabolic functions with the antibody response to the BBIBP-CorV vaccine	Observational study	China	Completed	The study population consists of adults who have received two doses of the inactivated SARS-CoV-2 vaccine (BBIBP-CorV; Sinopharm). The control group consists of patients who have a low antibody response to the BBIBP-CorV vaccine, defined as a neutralizing antibody titer of less than 30%.	gut microbes and their activities are linked to the BBIBP-CorV vaccine response, giving us clues for how to modulate gut microbes to improve the efficiency of current COVID-19 vaccines.	^44^
Dynamic changes in host immune system and gut microbiota are associated with the production of SARS-CoV-2 antibodies	Observational study	China	Completed	The study population consists of healthy adults who have received two doses of the inactivated SARS-CoV-2 vaccine (BBIBP-CorV; Sinopharm). The control group consists of patients who do not have dynamic changes in their host immune system and gut microbiota that are associated with the production of SARS-CoV-2 antibodies.	gut microbiota and body features affect antibody production the most. This means that gut microbiota is important for making SARS-CoV-2 antibodies in young healthy people.	^7^
Gut Microbiota Profile and Its Impact on Immunity Status in COVID-19 Vaccinated Cohorts	Observational study	China	uncompleted	The study population consists of adults who have received either the mRNA vaccine (BNT162b2; Pfizer-BioNTech) or the adenovirus vector vaccine (ChAdOx1 nCoV-19; Oxford-AstraZeneca) against COVID-19.	Not available	ClinicalTrials.gov Identifier:NCT04980560
Modulation of Gut Microbiota to Enhance Health and Immunity	Interventional study	China	uncompleted	The study population consists of healthy adults who have no history of chronic diseases or gastrointestinal disorders. The control group receives a placebo for 12 weeks, with no effect on the gut microbiota or the health and immunity of the host.	Not available	ClinicalTrials.gov Identifier: NCT04884776
Modification of the COVID-19 Vaccine Response by an Intervention on the Intestinal Flora	Interventional study	Canada	uncompleted	The study population consists of adults who have received two doses of the mRNA vaccine (BNT162b2; Pfizer-BioNTech) against COVID-19. The control group receives a placebo supplement containing maltodextrin for 12 weeks, with no effect on the intestinal flora or the immune response to COVID-19 vaccination.	Not available	ClinicalTrials.gov Identifier: NCT05195151

## Potential mechanisms by which the gut microbiota could regulate COVID-19 vaccines efficacy

The role of the gut microbiota in immune responses to COVID-19 vaccinations has yet to be determined. There are several proposed pathways that may explain this relationship, such as the alteration of B cell responses by microbial metabolites, the natural adjuvant hypothesis, and microbiota-encoded epitopes that are similar to COVID-19 vaccine antigens. This paper will analyze the available evidence for and against the proposed mechanisms. It is likely that the gut microbes can influence COVID-19 vaccines response in various ways; however, the complexity of these pathways and their dependence on the specific composition of the gut microbiota in different contexts may explain why it has been difficult to fully understand them.

## Innate lymphocytes may recognize increased opportunistic gut pathogens and intensify gut pro-inflammatory responses

**Figure 2.**
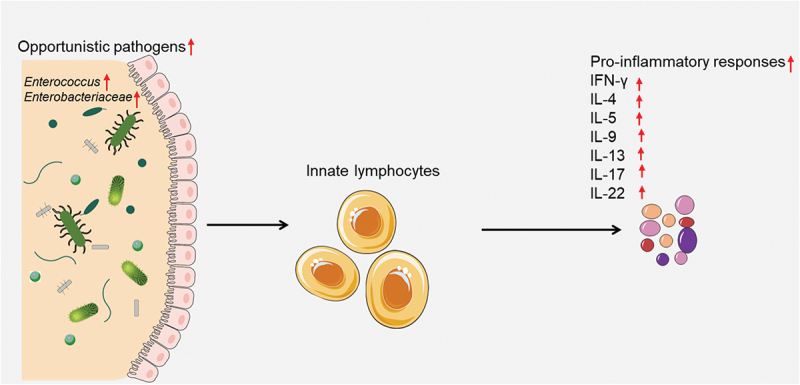
Innate lymphocytes recognize increased opportunistic gut pathogens and intensify gut pro-inflammatory responses.

Innate lymphocytes, comprising of ILC1, NK cell, ILC2, ILC3, LTi and innate-like cells like NKT, MAIT and γδ T cells, are particularly responsive to the gut microbiota due to their localized positioning and prompt primary responses^47,48^. These cells can recognize increased opportunistic gut pathogens and intensified gut pro-inflammatory responses. Upon activation, innate lymphocytes can produce various effector molecules that modulate the inflammatory response and the adaptive immunity. For example, NK cells can produce interferon-γ (IFN-γ), tumor necrosis factor-α (TNF-α) and perforin that can kill infected or transformed cells and enhance macrophage and T cell functions. ILCs can produce different subsets of cytokines depending on their lineage and polarization, such as IFN-γ, IL-4, IL-5, IL-9, IL-13, IL-17, and IL-22 that can regulate inflammation, tissue repair and mucosal immunity. γδ T cells can produce IFN-γ, TNF-α, IL-17 and IL-22 that can modulate inflammation, antimicrobial defense, and epithelial barrier function^49^. Opportunistic gut pathogens are microorganisms capable of inducing disease in individuals with compromised immune systems. Innate lymphocytes may recognize an increase in opportunistic gut pathogens and an intensification of gut pro-inflammatory responses (Figure 2). Previous studies have demonstrated that opportunistic gut microbes, such as *Enterococcus* and *Enterobacteriaceae*, can modulate the immune response to vaccines by regulating the production of pro-inflammatory cytokines^50^. One study found that mice with a higher abundance of pro-inflammatory bacteria in their gut had a stronger immune response to vaccination than those with a lower abundance of pro-inflammatory bacteria^51^. The researchers also found that treatment with antibiotics or probiotics could modulate the immune response to vaccination by altering the composition of gut microbiota. Consequently, pro-inflammatory responses are essential for initiating and maintaining an effective immune response to various pathogens and vaccines. In summary, innate lymphocytes may recognize increased opportunistic gut pathogens and promote gut pro-inflammatory responses, as well as influence the effectiveness of COVID-19 vaccines.

Innate lymphocytes can recognize increased opportunistic gut pathogens and intensified gut pro-inflammatory responses. Upon activation, innate lymphocytes can produce various effector molecules that modulate the inflammatory response and the adaptive immunity.

## Depleted commensal symbionts negatively regulate the recruitment of immune cells

**Figure 3.**
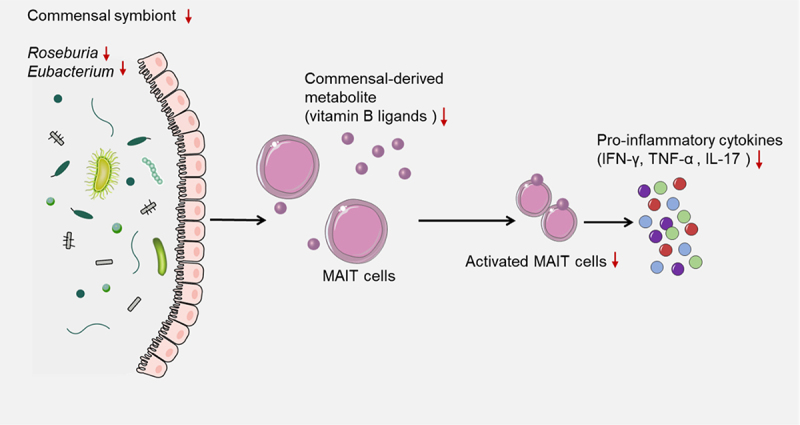
Depleted commensal symbionts negatively regulate the recruitment of immune cells.

Commensal symbionts are microorganisms that live in close association with a host organism and benefit from this relationship without causing harm to the host^52,53^. For example, the gut microbiota is a complex and dynamic community of commensal bacteria, fungi, archaea, and viruses that colonize the gastrointestinal tract and influence various aspects of host physiology and immunity. Depleted commensal symbionts could negatively influence the recruitment of immune cells, such as activated mucosal-associated invariant T (MAIT) cells, by affecting their activation, function, and migration^54,55^. MAIT cells are a subset of T cells that display innate-like qualities and can recognize bacterial ligands derived from vitamin B biosynthesis presented by the MR1 molecule, which are important for defense against bacterial and viral infections, including SARS-CoV-2^56,57^. One potential mechanism by which the gut microbiota can influence COVID-19 vaccine efficiency is through its impact on MAIT cells. Commensal bacteria that produce vitamin B ligands can directly stimulate MAIT cells and induce their proliferation and cytokine production. Therefore, depleted commensal symbionts, such as *Roseburia* and *Eubacterium*, may impair the activation and function of MAIT cells by reducing their stimulation, potentially resulting in decreased MAIT cell responses to SARS-CoV-2 infections, as well as reduced MAIT cell-mediated regulation of other immune cells^58^. The efficacy of a vaccine depends on the generation of robust and long-lasting immune responses against the target antigen. MAIT cells can contribute to vaccine efficacy by augmenting the innate and adaptive immune responses to vaccination. For example, MAIT cells can produce pro-inflammatory cytokines, such as IFN-γ, TNF-α, and IL-17, which can recruit and activate other immune cells, such as macrophages, neutrophils, and T cells. MAIT cells can also interact with B cells and promote their antibody production and class switching^59^. Moreover, MAIT cells can develop memory-like phenotypes and persist after infection or vaccination, thus providing rapid recall responses upon re-exposure to the same or related antigens^57^. In summary, depleted commensal symbionts could have a negative impact on the recruitment of immune cells, such as activated MAIT cells, potentially reducing the magnitude and quality of the immune responses to COVID-19 vaccination, resulting in lower levels of protection against infection or disease (Figure 3).

Commensal bacteria that produce vitamin B ligands can directly stimulate MAIT cells and induce their proliferation and cytokine production. Therefore, depleted commensal symbionts, such as Roseburia and Eubacterium, may impair the activation and function of MAIT cells by reducing their stimulation, potentially resulting in decreased MAIT cell responses to SARS-CoV-2 infections, as well as reduced MAIT cell-mediated regulation of other immune cells.

## Pattern recognition receptors are essential for the development of the immune system due to their innate sensing of the microbiota

**Figure 4.**
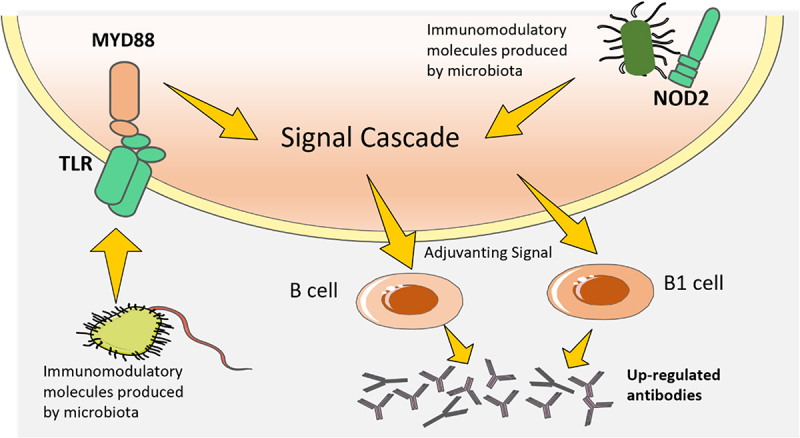
Pattern recognition receptors are essential for the development of the immune system due to their innate sensing of the microbiota.

The gut microbiota may influence the effectiveness of COVID-19 vaccines by providing natural adjuvants, such as aluminum salts, to increase, prolong, and improve antigen-specific immunological reactions (Figure 4). Adjuvants activate antigen-presenting cells, such as dendritic cells (DCs), through the recognition of microbial molecules by pattern recognition receptors (PRRs) such as Toll-like receptors (TLRs) and NOD-like receptors (NLRs)^60^. For instance, TLR5-mediated sensing of flagellin from intestinal microbes has been demonstrated to be necessary for optimal antibodies production in response to a non-adjuvanted coronavirus vaccine^61^. The results of a hemagglutination inhibition test further suggested that the magnitude of antibody titers was linked to the expression of TLR5 in blood mononuclear cells^62^. A recent study by another group did not find a strong correlation between TLR5 and the antibody responses to coronavirus or influenza vaccines in mice, which contrasts with the data previously mentioned^63^. The cause of these conflicting results has not fully investigated, but it is possible that the composition of the microbiota is a factor. Tlr5–/– mice had a significantly weaker antibody response to coronavirus vaccine when compared to non-littermate Tlr5+/+ mice, which showed a different intestinal microbial population. However, when compared to littermate Tlr5+/+ mice, which showed similarities in intestinal microbial population, the antibodies response to coronavirus vaccine was not attenuated.

It has been demonstrated that the activation of PRRs signaling pathways, which sense products made by microbes other than flagellin, can offer analogous adjuvant signal^64^. For instance, NOD2 is necessary for optimal responses to intranasal immunological reactions with cholera toxin and serum albumin, and the effect of the microbes on B-1 cells reactions to *Streptococcus* was linked to a key adaptor protein, MYD88, which is located downstream of various TLRs^65^. To investigate if the effects of intestinal microbes on vaccines response can also be mediated by other PRRs, further research is needed. Although it is known that microbial LPS sensed through TLR4 may boost vaccines reactions, it is yet to be seen if TLR4-mediated sensing of LPS produced by the intestinal microbial population affects vaccines immunity. This could be a complex issue, as different bacteria within the microbial population produce different varieties of LPS with varying levels of immunogenic potential.

It is probable that the microbiota’s capacity to act as a natural vaccine adjuvant is contingent on other elements, such as the quantity of immunomodulatory product and whether it is restricted to the intestine or escape into the environment^66^. When certain pathobionts, like the members of *Enterobacteriaceae* which produce LPS, proliferate due to inflammation or antibiotic use, the amount of LPS in intestine and periphery may be augmented, thus affecting the responses to vaccines administered at the same time^67^.

The gut microbiota may influence the effectiveness of COVID-19 vaccines by providing adjuvant signal to increase, prolong, and improve antigen-specific immunological reactions. Adjuvants provided by microbes can activate antigen-presenting cells, such as dendritic cells (DCs), through the recognition of microbial molecules by pattern recognition receptors such as Toll-like receptors (TLRs) and NOD-like receptors (NLRs).

## Antigen-presenting cell can be reprogrammed by microbiota

**Figure 5.**
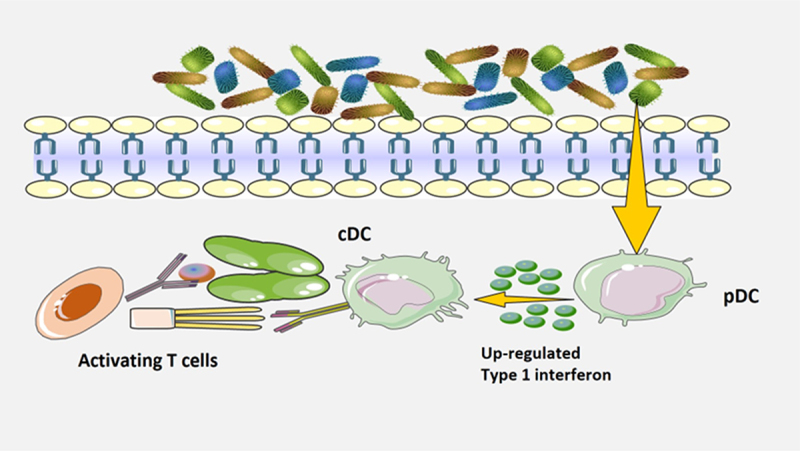
Antigen-presenting cells can be reprogrammed by microbiota.

DCs, which are antigen-presenting cells, are essential to present vaccines antigen to T cells and regulating the intensity, longevity, and quality of the resulting immunological reactions. PRRs are responsible for the primary function of immune cells, and more and more evidence suggest that the microbes may powerfully influence DC functions^41,66^, implying that the microbes might be used as a natural adjuvant for vaccines (Figure 5). Following immunization via intranasal administration of inactive cholera toxin, detection of the microbes by lung DCs through TLR-mediated processes resulted in an increase of gut-homing receptors α4β7 integrin and CC-chemokine receptor 9 in IgA+ B cells^68,69^. This cell migration from lung to intestine was found to provide immune responses to cholera toxin^70^. Intranasally immunizing mice(germ-free) with inactive cholera toxin has been observed to significantly reduce the production of antigen-specific IgA in intestine. Moreover, the decreasing of microbes by antibiotic can impede the amount of total IgA produced in the lungs of humans in intensive care units and mice models, thus increasing the chances of contracting SARS-CoV-2^71,72^.

In recent days, it has been demonstrated that the microbiota can control the type I interferons’ levels by plasmacytoid DCs, which leads to a particular metabolic and epigenomic state in conventional DCs, thus allowing them to more effectively initiate antigen-specific T cells response^15,73^. Furthermore, the role of microbes in regulating immune responses can be seen in its ability to boost vaccines response by influencing other antigen-presenting cells. An example of this is the enhancement of antibody responses to non-adjuvanted influenza vaccines due to the presence of microbes-produced flagellin.^74,75^. This effect was found to be independent of dendritic cells, and the vaccine’s ability to induce antibodies response was found to be reliant on macrophages; mice which had their macrophages removed were unable to create a detectable response one week after being immunized. Furthermore, research has shown that the microbes can affect how antigens are presented by intestinal epithelial cells; this has potential effects on the immune system’s reactions to oral vaccine. Additionally, intestinal microbes may also have a direct impact on both T cells and B cells^76^.

DCs, which are antigen-presenting cells, are essential to present vaccines antigen to T cells and regulating the intensity, longevity, and quality of the resulting immunological reactions. PRRs are responsible for the primary function of immune cells, and more and more evidence suggest that the microbes may powerfully influence DC functions, implying that the microbes might be used as a natural adjuvant for COVID-19 vaccines. (plasmacytoid dendritic cell, pDC; conventional DC, cDC).

## Microbiota-derived metabolites modulate immune responses

**Figure 6.**
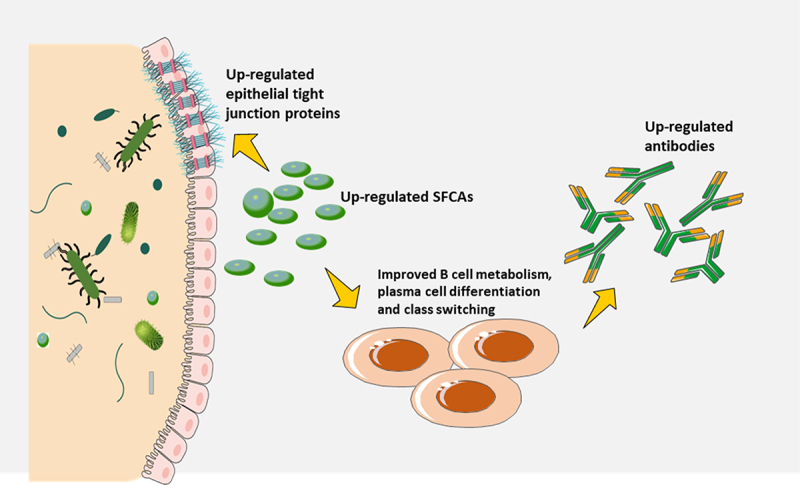
Microbiota-derived metabolites modulate immune responses.

The gut microbiota not only produces molecules that can be sensed by PRRs, but also many metabolites that can influence immune responses (Figure 6). Short-chain fatty acids (SCFAs) like butyrate, propionate, and acetate, which are the major metabolites of intestinal microbial fermentation, are among the most studied of these microbiota-derived metabolites^77,78^. Studies have shown that SCFAs can enhance fatty acid production, glycolysis, and oxidative phosphorylation in B cells, providing the energy required to sustain optimal homeostatic antibodies response and antibodies generated in response to infections^79^. The results in this study also indicated that SCFAs could increase the protein expressions related to plasma cell class switching and differentiation. However, another research suggested that SCFAs could suppress antibody responses to ovalbumin administered through the stomach and autoantibody responses^80,81^. Due to the conflicting findings mentioned above, further research is necessary to determine the effect of SCFAs on antibodies reactions to different vaccines.

Many metabolites derived from the microbiota possess immunomodulatory properties, such as tryptophan metabolites, SCFAs, and secondary bile acids. For instance, a study has demonstrated that antibiotic treatment can markedly decrease the secondary bile acids production, and this decrease was associated with an increased inflammatory response in people who had been vaccinated against the SARS-CoV-2^82^. Earlier, it was mentioned that intestine barrier integrity may be strengthened by microbes-derived metabolites, which may in turn indirectly modulate immunological reactions to vaccination, thus potentially preventing microbial molecules from escaping and intensifying parenteral vaccine responses^83,84^. Recent studies have suggested that metabolites generated by certain intestinal microbial species, such as SCFAs, may help maintain the integrity of the gut barrier by increasing the amount of epithelial tight junction proteins. This benefit is likely to affect the reactions to parenteral vaccines, as tight junction proteins are involved in regulating the permeability of the intestinal epithelium and preventing the translocation of pathogens and antigens^68^. In order to evaluate the immunomodulatory effects of these metabolic products on other immune cells that are accountable for reactions to vaccination, like T cells and DCs, further research must be done. SCFAs have strong effects on T cells in other circumstances, however, it is yet to be determined if SCFAs can manage vaccines-induced T cell-mediated immune response.

The gut microbiota not only produces molecules that can be sensed by PRRs, but also many metabolites that can influence immune responses. SCFAs may enhance fatty acid production, glycolysis, and oxidative phosphorylation in B cells, providing the energy required to sustain optimal homeostatic antibodies response and antibodies generated in response to COVID-19 infections. In addition, metabolites generated by certain intestinal microbial species, such as short-chain fatty acids (SCFAs), may help maintain the integrity of the gut barrier by increasing the amount of epithelial tight junction proteins, which is likely to affect the reactions to parenteral vaccines.

## Antigens encoded by microbiota that are capable of cross-reactivity

**Figure 7.**
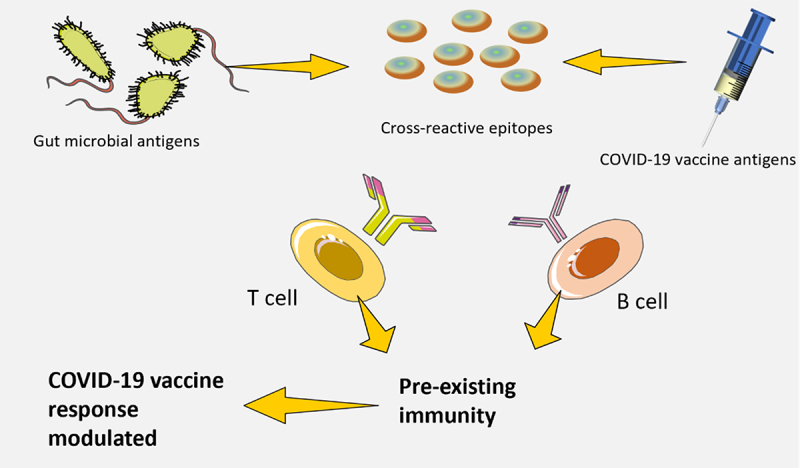
Antigens encoded by microbiota that are capable of cross-reactivity.

It has been observed in the past that memory T cell (CD4+), which are specific to antigens encoded by pathogens, can be found in people who have never been infected by those pathogens^34,85^. This could be due to T cell receptor (TCR) cross-reactivity occurring in response to environmental antigens, especially those produced by the intestinal microbes (Figure 7). Healthy individuals have a large number of T cells (CD4+) in their blood and tissues that are reactive to the intestinal microbiota. Bioinformatic analysis suggests that there is a considerable overlap between the TCR epitope repertoires of the human proteome and the microbes, thus, likely, with antigens produced by vaccines^86,87^. Evidence is growing to suggest that cross-reactive T cells may influence the body’s immune response to pathogens^88^. This effect can either reduce or increase the immunogenic potential of the microbe’s antigenic epitope. Studies have also found that the presence of cross-reactive T cells before exposure to the influenza vaccine is linked to a stronger immune response^89^.

The origin of these cross-reactive T cells has yet to be identified through these studies; however, other research has identified T cells that are able to recognize influenza virus-derived epitopes and peptides originating from the microbiota. Recently, it was discovered that epitopes of a bacteriophage protein produced by *Enterococcus* hirae and MHC class I-restricted tumor antigens are cross-reactive. In addition, animals colonized with Enterococcus hirae containing the bacteriophage demonstrated improved reactions to immunotherapy^90^. The potential for the gut microbiota to influence responses to vaccination by activating T cells or B cells that cross-react with vaccines antigen remains unclear. Further research is needed to explore this possibility.

Memory T cell (CD4+), which are specific to antigens encoded by pathogens, can be found in people who have never been infected by those pathogens. This could be due to T cell receptor (TCR) cross-reactivity occurs in response to environmental antigens, especially those produced by the intestinal microbes. Cross-reactive T cells may influence the body’s immune response to COVID-19. This effect can be either to reduce or increase the immunogenic potential of the microbe’s antigenic epitope.

## Targeting gut microbiota as a potential intervention to enhance the effectiveness of COVID-19 vaccines

**Figure 8.**
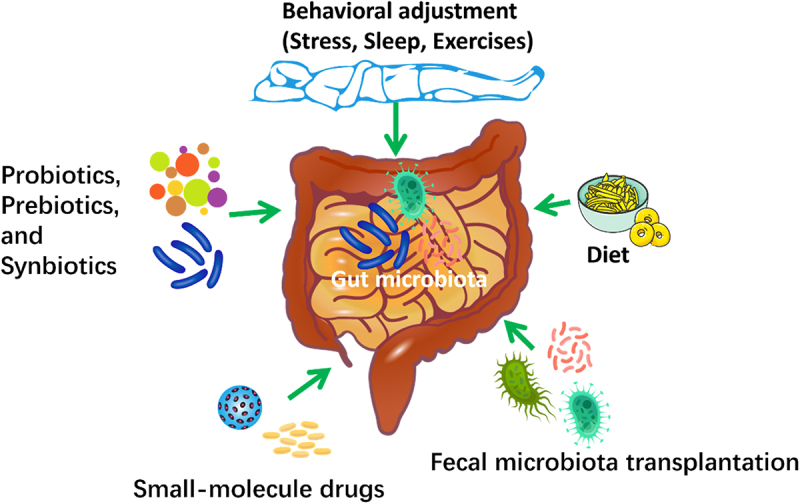
Potential gut microbiota-targeted interventions to improve the efficacy of COVID-19 vaccines.

Investigations into the use of gut microbiota-targeted interventions, such as diet, probiotics, prebiotics and synbiotics, behavioral adjustment, fecal microbiota transplantation (FMT), and small-molecule agents that modulate certain microbial processes, are widely applied to enhance the effectiveness of COVID-19 vaccines. It is possible that microbiota-targeted interventions may be beneficial even if they do not completely restore the intestinal microbes to a “healthy” condition, due to the possible effects of intestinal barrier integrity and the movement of microbes’ products on reactions to COVID-19 vaccines^91,92^. The gut microbiota-targeted interventions could be used to modulate the gut microbiota and enhance COVID-19 vaccine immunogenicity as well as reduce adverse events (Figure 8). However, more research is required to confirm the causal relationship between gut microbiota and COVID-19 vaccine response, elucidate the underlying mechanisms, and assess the efficacy and safety of microbiota-based interventions.

Investigations into the use of gut microbiota-targeted interventions, such as diet, probiotics, prebiotics and synbiotics, behavioral adjustment, fecal microbiota transplantation (FMT), and small-molecule agents that modulate certain microbial process, are widely explored to enhance the effectiveness of COVID-19 vaccines.

## Diet

The stability, diversity, and composition of the gut microbiota can be impacted by diet, indicating that dietary strategies may be used therapeutically to manipulate it. However, diet-induced changes in the gut microbiota appear to be temporary and may depend on the duration of dietary interventions^93^. Different types of diets can have different effects on the gut microbiota. For example, a diet that is high in animal protein and fat can increase the abundance of *Bacteroides*, *Alistipes* and *Bilophila* species, which are associated with inflammation and metabolic disorders. A study by Jordan et al. demonstrated that a diet high in plant-based fiber increased the abundance and activity of glycan-degrading bacteria in the gut, potentially enhancing the immune response to vaccines by stimulating trained immunity^94^. Therefore, maintaining a healthy and balanced gut microbiome through dietary regulation may have positive impacts on the efficacy of COVID-19 vaccination.

## Prebiotics, probiotics, and synbiotics

Probiotics and prebiotics are potential gut microbiota-targeted interventions to improve the efficacy of vaccines because they can modulate the immune system and influence the gut microbiome composition. As we have mentioned above, several interventional clinical studies have demonstrated that probiotics and prebiotics might regulate COVID-19 vaccines efficacy and safety by modulating the gut microbiota. In addition, some studies have indicated that synbiotics, a combination of prebiotics and probiotics, may also enhance the production of antibodies and cytokines after vaccination^70,91,94^. However, there is not much data on using synbiotics for improving COVID-19 vaccines efficacy specifically. Most of the data are from studies on other vaccines, such as rotavirus, influenza, or polio vaccines. For example, A study by Kwon et al. showed that a synbiotic containing *Lactobacillus* plantarum DR7 improved antibody titers against influenza virus after vaccination in healthy adults^95^. Another study by Zhang et al. showed that a synbiotic containing *Bifidobacterium* animalis subsp. lactis BL-99 enhanced immunogenicity of oral polio vaccine in infants^96^. A recent study by Spacova et al. showed that a synbiotic containing *Bifidobacterium* longum increased serum IgG levels against rotavirus after vaccination in children^97^. These studies suggest that synbiotics may have a positive effect on vaccine efficacy by modulating the gut microbiota and enhancing the immune response to antigens. However, more research is needed to confirm this effect for COVID-19 vaccines specifically. In addition, future studies should focus on determining the optimal dose, duration, mode of administration (stand-alone or add-on), timing, and strain-specificity of probiotics, prebiotics and synbiotics for enhancing COVID-19 vaccine efficacy. Furthermore, the mechanisms underlying their immunomodulatory effects are also not fully understood. Therefore, further research is warranted to elucidate how these substances can be used as adjuvants or adjuncts in COVID-19 vaccination strategies.

## Behavioral adjustment

One possible way that behavioral adjustment could influence the gut microbiota to improve the efficacy of COVID-19 vaccines is by reducing stress, which is known to affect both the immune system and the composition of the gut microbes. Stress affects the gut microbiota by activating the hypothalamic-pituitary-adrenal (HPA) axis, which is the main neuroendocrine system that mediates the stress response. The HPA axis modulates the secretion of various hormones, such as cortisol and catecholamines, that can alter the composition and function of the gut microbes, as well as their interactions with the host immune system and metabolism^98^. For example, a study by Jordan et al. suggested that stressful events during pregnancy could have a negative impact on maternal gut microbiota composition and diversity, which could be transmitted to infants via vertical transmission^94^. This could affect the development of the infant’s immune system and their response to vaccines later in life. Another study by Bailey et al. found that social disruption stress altered both microbial community structure and community membership in mice^99^. This was associated with increased inflammation and impaired antibody production after vaccination. However, the antibiotic-treated mice did not show an increase in IL-6 and MCP-1 when exposed to social disruption stress. SCFAs also have a critical role in regulating the decline of immune function related to anxiety or stress. SCFAs are microbial metabolites known to have anti-inflammatory and immunoregulatory effects, and they can also enhance vaccine-induced immunity^100^. A recent study by Dalile et al. showed that chronic psychosocial stress reduced fecal SCFAs levels and SCFAs supplementation restored stress-induced immunosuppression in healthy men^101^.

A good-quality sleep is known to have positive effects on our immune system and the gut microbiota, both of which are involved in vaccine responses. Sleep can affect the gut microbiota by modulating the circadian rhythm, which is the biological clock that synchronizes various physiological processes with the day-night cycle. The circadian rhythm regulates the activity and composition of the gut microbes, as well as their interactions with the host immune system and metabolism. A study by Voigt et al. found that “sleep fragmentation reduced bacterial richness and diversity” and “altered bacterial community composition” in female populations^16^. This was associated with increased inflammation and impaired antibody production after vaccination. Exercise is another potential behavioral factor that may influence the intestinal microbes and enhance the efficacy of COVID-19 vaccines. Previous studies have demonstrated that exercise can modulate the composition and diversity of the intestinal microbes, as well as its metabolites and functions^102^. In addition, the intestinal microbes may also affect exercise-induced inflammation, oxidative stress, muscle damage, and immune responses^103^. In a recent study, swimming exercise has been shown to improve depressive-like behavior and boost immunity through its anti-inflammatory effects, as well as by restoring the balance of gut bacteria, such as *Escherichia* coli and *Lactobacilli*^104^.

The relationship between behavioral adjustment, intestinal microbes, and COVID-19 vaccine efficacy is complex and multifaceted, and may depend on many factors such as age, genetics, environment, nutrition, infection history, vaccine type, and dose. Therefore, more research is needed to understand how these factors interact and how they can be modulated to optimize COVID-19 vaccine responses.

## Fecal microbiota transplantation (FMT)

FMT, or fecal microbiota transplantation, is a procedure that works to restore the gut microbiota of the recipient to a normal state in order to provide a therapeutic benefit. FMT has been applied in various gastrointestinal and extra-gastrointestinal diseases, including ulcerative colitis (UC) and *Clostridium* difficile infection (CDI)^105^. FMT may also influence the immune system and the efficacy of vaccines, but the mechanisms are not fully understood. According to one study, FMT from an effective donor (who induced remission in 100% of UC patients) was associated with higher donor microbiota species evenness, stability, engraftment, and functional differences compared with a less effective donor (who induced remission in 36% of UC patients)^106^. The effective donor had higher abundance of some beneficial bacteria, such as *Faecalibacterium* prausnitzii, *Roseburia* intestinalis and *Akkermansia* muciniphila. These bacteria may modulate the immune system and enhance the response to vaccines.

Another study found that FMT from healthy donors improved the clinical symptoms and reduced the inflammation in mice with experimental autoimmune encephalomyelitis (EAE), a model of multiple sclerosis^107^. FMT also altered the gut microbiota composition and increased the abundance of some anti-inflammatory bacteria, such as *Bacteroides* fragilis and *Lactobacillus* reuteri. In addition, FMT also regulated the expression of some genes related to immune response, inflammation, and intestinal barrier function.

## Small-molecule drugs

Small molecule drugs, defined as any organic compound with low molecular weight, offer distinct advantages as therapeutics. These advantages include the ability to be administered orally and to pass through cell membrane to reach intracellular target. Several small-molecule drugs have been found to affect the composition and function of the gut microbiota, which, in turn, may alter the host immune system and vaccine efficacy. For example, a small molecule bioactive peptide R7I could affect the composition of gut microbiota in mice model^108^. The R7I treatment drastically lowered the presence of *Clostridia* and increased the dominance of *Odoribacteraceae*, an efficient isoalloLCA-synthesizing strain that was able to ward off potential pathogens. Furthermore, this study discovered that R7I lessened the buildup of negative organic acid metabolites. In general, R7I exhibit significant immunomodulatory effects in mice and may be a potential gut microbiota-targeted agent to enhance COVID-19 vaccination. Metformin is a small-molecule drug with multiple pharmacological functions. A study by Li et al. found that metformin changed the gut microbiota composition and function in healthy volunteers, and increased the abundance of *Akkermansia*, which are associated with anti-inflammatory effects and improved metabolic health^109^. Interestingly, this research also indicated that metformin enhanced the antibody responses to influenza vaccine in humans. A recent study by Volpe et al. has developed a novel small molecule inhibitor which can prevent gut bacterial genotoxins production in C57BL/6J mice^110^. Accumulating evidence has indicated that one of the main purposes of bacterial genotoxins is to disrupt the immune response of the host cells they affect, as they are capable of inducing DNA damage. Thus, this novel small molecule inhibitor might be a potential gut microbiota-targeted agents to improve the efficacy of COVID-19 vaccines.

Although the aforementioned gut microbiota-targeted interventions may improve the efficacy of COVID-19 vaccines, there are some possible limitations and challenges to these interventions. For example, the efficacy and safety of probiotics are not well established and depend on various factors, such as the strain, dose, formulation, delivery method, host characteristics and disease context. Moreover, probiotics may not colonize the gut effectively or may interact negatively with the resident microbiota or the immune system. Therefore, more rigorous clinical trials and mechanistic studies are needed to evaluate the optimal use of probiotics for different indications^40^. Dietary interventions also face several challenges, such as the variability in individual dietary habits, preferences and adherence, as well as the influence of genetic, environmental and lifestyle factors on the gut microbiota and health. Moreover, dietary interventions may have unintended consequences, such as nutrient deficiencies, weight changes or metabolic disorders. Therefore, more personalized and holistic approaches are needed to optimize dietary interventions for different goals and contexts^65^. In addition, FMT also poses several challenges, such as the variability in donor selection and screening, fecal preparation and administration, recipient response and outcome assessment. Moreover, FMT may carry the risk of transmitting infections or diseases from the donor to the recipient, or causing adverse events such as diarrhea, abdominal pain, fever, or immune reactions. Therefore, more standardized protocols and regulations are needed to ensure the quality and safety of FMT and to monitor its long-term effects. Some of the major ethical issues with FMT should also be carefully considered, such as informed consent and the vulnerability of patients, determining what a ‘suitable healthy donor’ is, safety and risk, commercialization and potential exploitation of vulnerable patients, and public health implications^5^.

## Discussions and future perspectives

Natural immunity to COVID-19 infection is determined by the persistence and functionality of immune memory cells and antibodies that are generated after primary infection. The duration of natural immunity can vary depending on several factors, such as the severity and duration of infection, the viral load and variant, the age and health status of the individual, and the exposure to subsequent infections or vaccines^111^.

Several studies have estimated the duration of natural immunity to COVID-19 infection by measuring the levels of antibodies and memory cells in convalescent individuals over time. These studies have reported different results, ranging from a few months to more than a year, depending on the methods and populations used. For example, a study by Moore et al. showed that SARS-CoV-2-specific memory B cells were detectable up to 12 months after infection in 87% of convalescent individuals, while a study by Ahluwalia et al. showed that SARS-CoV-2-specific memory T cells were detectable up to 8 months after infection in 95% of convalescent individuals^112,113^. Natural immunity to SARS-CoV-2 infection is not only mediated by antibodies, but also by memory B cells and T cells, which are crucial for long-term protection and recall responses. Memory B cells and T cells are generated after primary infection or vaccination and can persist for months or years in the absence of antigen stimulation. Upon re-exposure to the same or a similar antigen, memory B cells and T cells can rapidly reactivate and differentiate into effector cells, such as plasma cells and cytotoxic T cells, that can produce high-affinity antibodies and eliminate infected cells, respectively^114^. Memory B cells and T cells have several advantages over antibodies in providing long-term immunity to SARS-CoV-2 infection. First, memory B cells and T cells can undergo somatic hypermutation and affinity maturation, which enable them to produce antibodies with higher specificity and potency against the virus^115^. Second, memory B cells and T cells can recognize a broader range of viral epitopes, including those that are conserved or hidden from antibody recognition, which enhance their ability to cope with viral variants^116^. Third, memory B cells and T cells can adapt to different tissues and microenvironments, such as the respiratory mucosa and the lymph nodes, which facilitate their localization and function at the sites of infection^115^.

The evidence presented in previous studies strongly indicates that the gut microbiota has an influence on both T cell and B cell responses to COVID-19 vaccination. For example, a recent clinical study compared the T cell and B cell responses to BNT162b2 vaccination in health care professionals with or without previous COVID-19 infection^117^. Their results speculated that gut microbiota may play a pivotal role in regulating the balance between innate and adaptive immunity. Another study by Sureshchandra et al. used scRNA-Seq and functional assays to compare the B cell and T cell repertoires following two doses of mRNA vaccine with responses observed in convalescent individuals with asymptomatic COVID-19^118^. They revealed activated CD4+ T cells, enrichment of spike-specific B cells, and robust antigen-specific polyfunctional CD4+ T cells response after COVID-19 vaccination. As more observational studies are conducted in different populations, associations between certain bacterial phyla and families in intestinal microbes and immunological reactions to COVID-19 vaccines have been identified; however, it is yet to be determined if these associations are causal. To prove these associations, it is essential to identify the exact strains or species of microbes responsible for these effects, as well as to understand the mechanisms behind them. Previous cohort studies have not been able to resolve the microbiota at the species and strain level, as they only used 16S rRNA sequencing instead of shotgun metagenomics^119,120^. Moreover, interventional studies that tested how antibiotics or probiotics affect vaccination reactions have been too small to detect significant effects of the microbiota; however, they indicate that the microbiota may influence antibodies reactions more in people with lower preexisting immunity, and the innate immune responses or metabolome can be greatly impacted by the gut microbiota^40,121^.

As we have discussed above, many factors can affect the COVID-19 vaccine efficacy and shape the gut microbiota and baseline immunity. However, it is exceptionally difficult to untangle these intricate interdependent relationships in clinical cohort studies. To study these complex relationships, we need to use methods that combine different types of data from the immune system and the COVID-19 vaccine with proper statistical methods to account for factors that might interfere with the results. Another way to unravel the complex connections between these factors is to conduct carefully designed preclinical studies. These studies can help to control for the various factors by strictly modulating the diet and environment, as well as matching the age and sex of the animal models.

In this review, possible immunological mechanisms have been proposed to explain how the intestinal microbes may regulate immune responses to COVID-19 vaccination. In addition, we have summarized various potential gut microbiota-targeted interventions to improve the efficacy of COVID-19 vaccines. However, these mechanisms and interventions remain hypothetical due to the limited sample sizes and the conflicting evidence among some studies. Further research is required to clarify these potential mechanisms and interventions in various settings. In addition, it is more and more accepted that COVID-19 vaccine not only trigger specific immune response but also have strong nonspecific effects on immunological reactions to other pathogens. The role of the gut microbiota in the nonspecific effects of COVID-19 vaccines is, as far as we know, almost totally unknown. Considering the growing recognition of the potential significance of these effects, studies in this area should be given high priority.

This comprehensive review on the interaction between gut microbiota and COVID-19 vaccines may have important implications for future research, clinical practice, and public health. Firstly, it may provide new insights into the mechanisms and modulators of COVID-19 vaccine-induced immunity, as well as the potential role of the gut microbiota in mediating vaccine efficiency and safety. Future research should aim to elucidate the causal relationships and molecular pathways underlying the interactions between the gut microbiota and the immune system in response to different types of COVID-19 vaccines, such as inactivated, mRNA, viral vector or protein subunit vaccines. Secondly, this review may have potential applications for clinical practice, such as the use of microbiota profiling or biomarkers to predict or monitor vaccine responses, or the use of microbiota-targeted interventions to modulate the gut microbiota and improve vaccine outcomes. For example, gut microbiota composition has been associated with SARS-CoV-2 vaccine immunogenicity and adverse events in adults who have received the inactivated vaccine (CoronaVac; Sinovac) or the mRNA vaccine (BNT162b2; BioNTech; Comirnaty). Thus, microbiota analysis could help identify individuals who are likely to have low or high vaccine responses, or who are prone to experience adverse events, and tailor personalized vaccination strategies accordingly. At last, our review may also have relevance for public health, such as the consideration of individual and population-level factors that affect the gut microbiota and COVID-19 vaccine responses, or the implementation of dietary and lifestyle interventions to optimize the gut microbiota and vaccine efficacy. For example, age, sex, ethnicity, genetics, diet, medication use, comorbidities and environmental exposures can influence the gut microbiota composition and diversity, as well as the immune response to vaccination. Therefore, public health policies and programs should consider these factors and address the potential disparities and inequalities in vaccine access and response among different groups of people.

## Abbreviations

COVID-19: coronavirus disease 2019
SARS-CoV-2: Severe Acute Respiratory Syndrome Coronavirus 2
WHO: World Health Organization
EUL: Emergency Use Listing
SCFAs: short-chain fatty acids
TLRs: Toll-like receptors
pDC: plasmacytoid dendritic cell
cDC: conventional dendritic cell
FMT: fecal microbiota transplantation
UC: ulcerative colitis
CDI: Clostridium difficile infection
VLP: virus-like particles
PTSD: post-traumatic stress disorder
HPA: hypothalamic-pituitary-adrenal
